# Supplementary material for: Cryo-correlative light and electron tomography of dopaminergic axonal varicosities reveals non-synaptic modulation of cortico-striatal synapses
Source: Nat Commun. 2025 Dec 13;16:11467. doi: 10.1038/s41467-025-66355-x (PMC12749485; doi:10.1038/s41467-025-66355-x)
Supplement: Supplementary file 1 — Supplementary Information [file 41467_2025_66355_MOESM1_ESM.pdf]

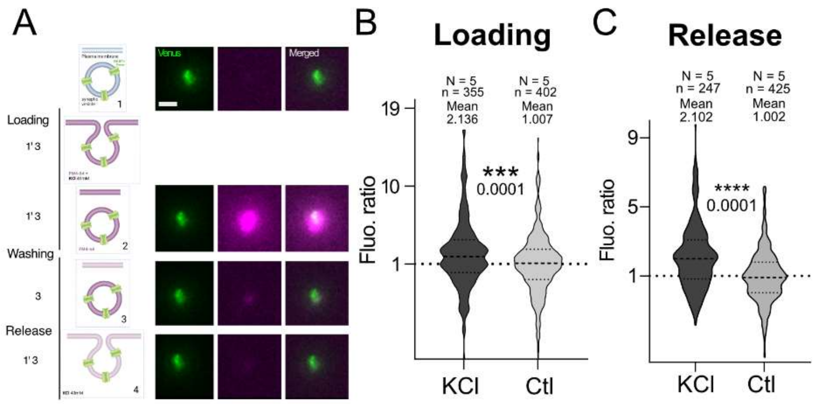

Figure S1: Synaptic vesicle cycling in synaptosomes measured with FM4-64 uptake and release. **A**, Scheme of the principle of FM assay in synaptosomes. A first image is acquired before adding the dye and corresponds to the background (1). FM4-64 is loaded into vesicles by triggering a first exo-endocytic cycle with 40 mM KCl and a second image is taken (2). After washing with HBK, only cycling vesicles remain stained by FM4-64 monitored by a third image (3). A second stimulation with 40 mM KCl induce release of the dye and is followed by washing with HBK and acquisition of a final image (4). A control experiment is performed without KCl depolarization for loading to monitor unspecific dye labelling. Scale bar 500 nm. **B**, Violin plot showing the distribution of loading values (acq. 3 intensity - acq. 1 intensity) normalized to the control. On average the loading signal is 2.136 times higher upon stimulation with KCl (dark grey) which correspond to synaptosomes that have loaded the dye (Mann-Whitney; p-value < 0.0001). **C**, Violin plot showing the distribution of release values (acq. 3 – acq. 4) after stimulation (dark grey) normalized to the control (light grey). Destaining averages 2.102 times higher than the mean value of control (Mann-Whitney; p-value < 0.0001).

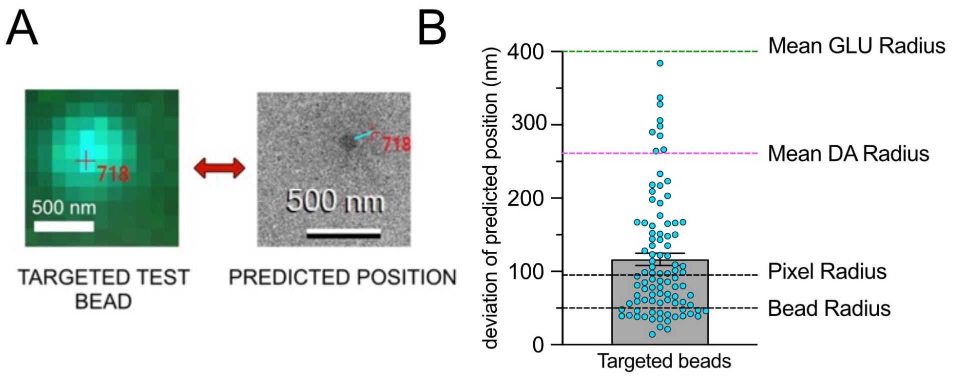

Figure S2: Pointing precision of the alignment between fluorescence and electron microscopy
A, Representative images of a fluorescent fiducial bead that has not served for the correlation, in cryo-fluorescence microscopy (left) and in cryo-EM (right). The red cross (718) indicates the targeted position on the fluorescent image and the predicted position obtained on the cryo-EM after transformation. The cyan line shows the distance between the predicted position and the actual bead **B**, Histogram showing the deviation of the predicted positions in nanometers for beads that have not been used for the transformation. The average distance is 117 nm which corresponds to 42% of the mean DA radius and 31% the mean GLU radius (n = 95).

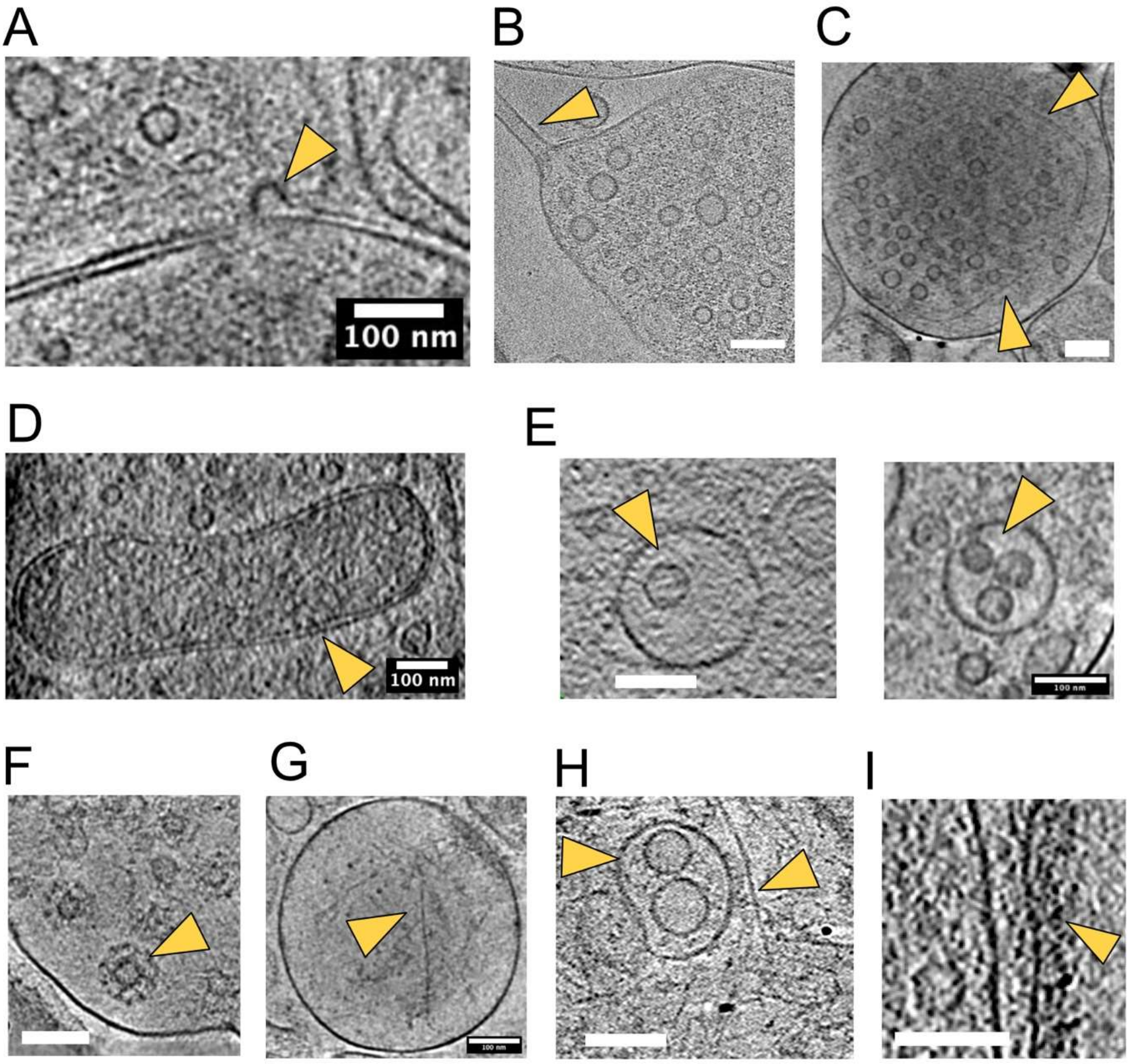

Figure S3: Examples of structures and organelles found in GLU synaptosomes (A-F) and post-synaptic elements (G-I). Yellow arrows point to the organelles of interest in all panels. **A**, Example of a membrane invagination, either an exocytic or endocytic event. **B**, Example of a narrow membrane tubule on one side of the GLU synaptosome. Perhaps a remaining axonal part filled with microtubules. **C**, Example of filaments surrounding synaptic vesicles. **D**, Example of a mitochondrion. **E**, Examples of multivesicular bodies. **F**, Example of clathrin-coated vesicles. **G**, Example of actin filaments in the PSE. **H**, Examples of a multivesicular body and a dense actin filaments network in a PSE. **I**, Example of a post-synaptic density. Scale bars 100 nm.

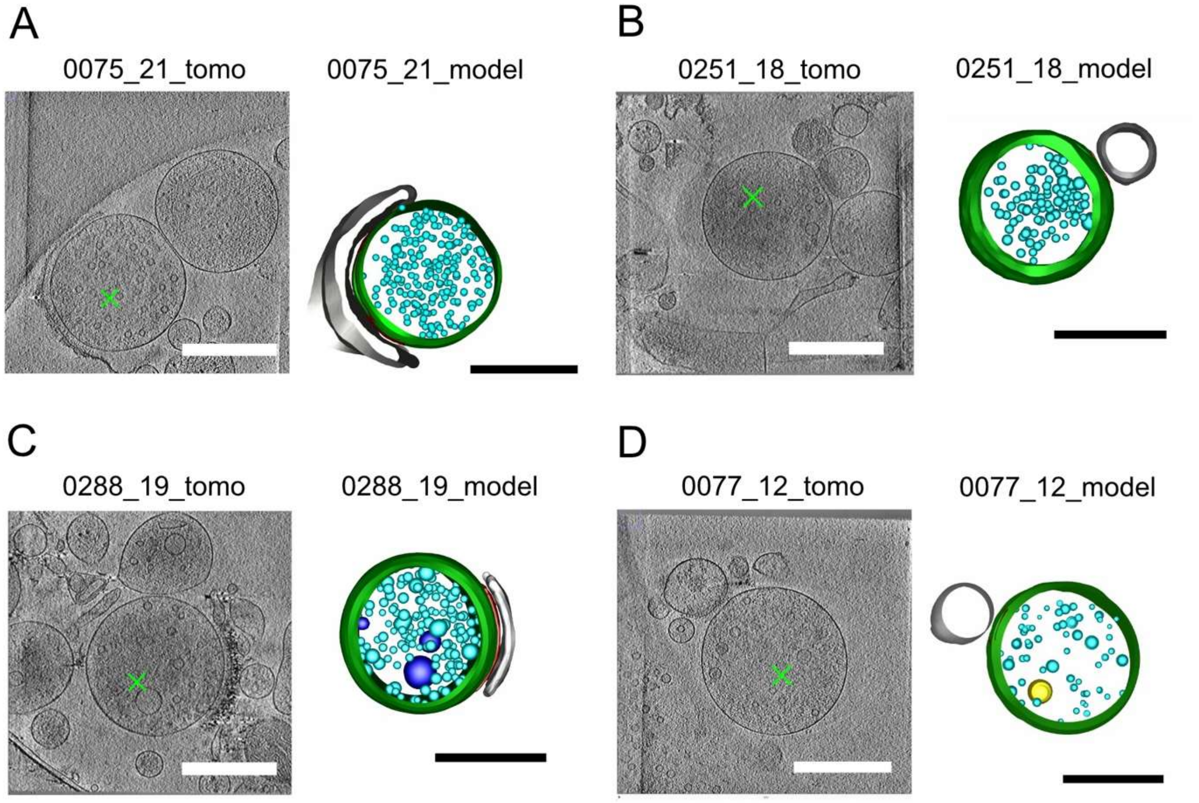

Figure S4: Gallery of segmented GLU synaptosomes. A, Example of a tomogram (numbered 0075_21) showing a GLU synaptosome (green cross, left) and the corresponding 3D model (right). The plasma membrane is represented in green, the post-synaptic element in light grey and synaptic vesicles in cyan **B,** Similar example for the tomogram numbered 0251_18. **C**, Similar example for tomogram 0288_19. Multivesicular bodies are drawn in dark blue. **D,** Similar example for tomogram 0077_12. Large vesicle is shown in yellow. Scale bar: 500 nm.
For the 3D models, the color coding is the same as in Figure 2 and 3.

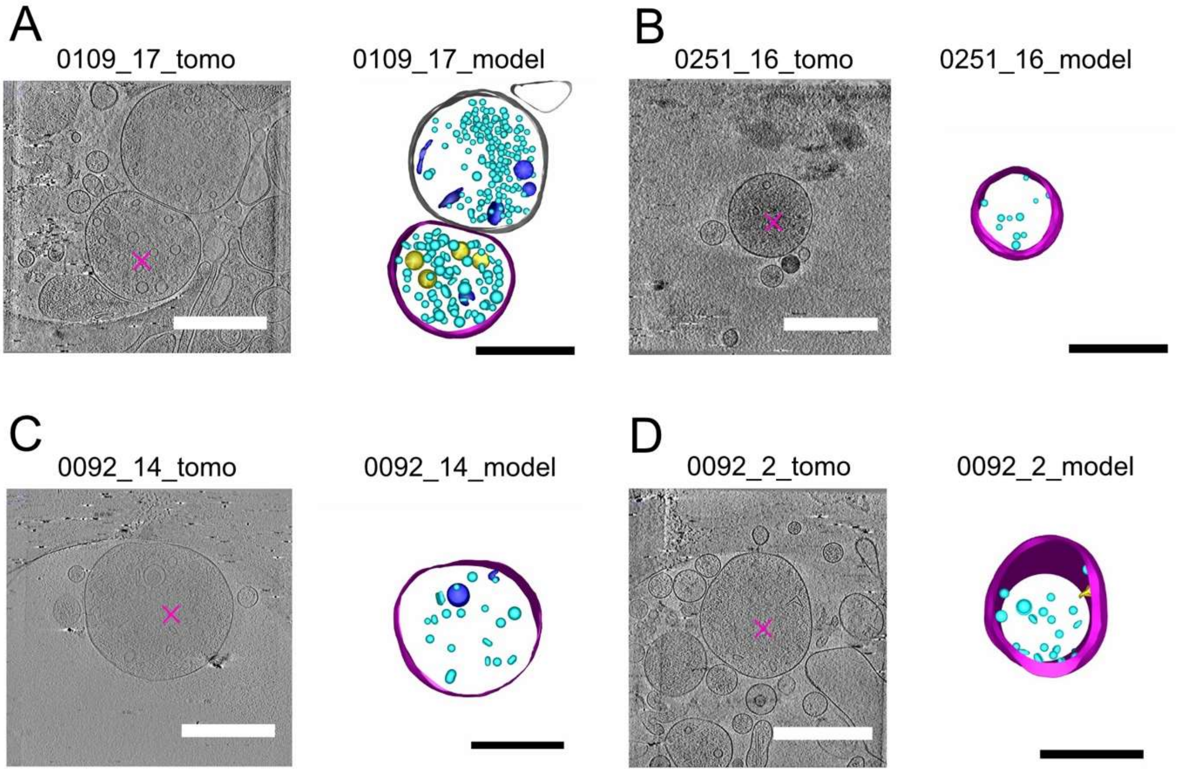

Figure S5: Gallery of segmented DA synaptosomes. A, Example of a tomogram (numbered 0109_17) showing a DA synaptosome (magenta star, left) and the corresponding 3D model (right). The DA element adheres to another element resembling a GLU synapse with many SVs and a clear PSE (top right). However, this tomogram was obtained in a mouse in which only DA neurons are fluorescent (DAT-Cre injected with AAV-Flex-NeonGreen), so the identity of the adhering structure could not be confirmed with fluorescence. **B,** Similar example for tomogram 0251_16 **C**, Similar example for 0092_14 **D,** Similar example for 0092_2. Scale bars: 500 nm. For the 3D models, the color coding is the same as in Figure 2 and 3.

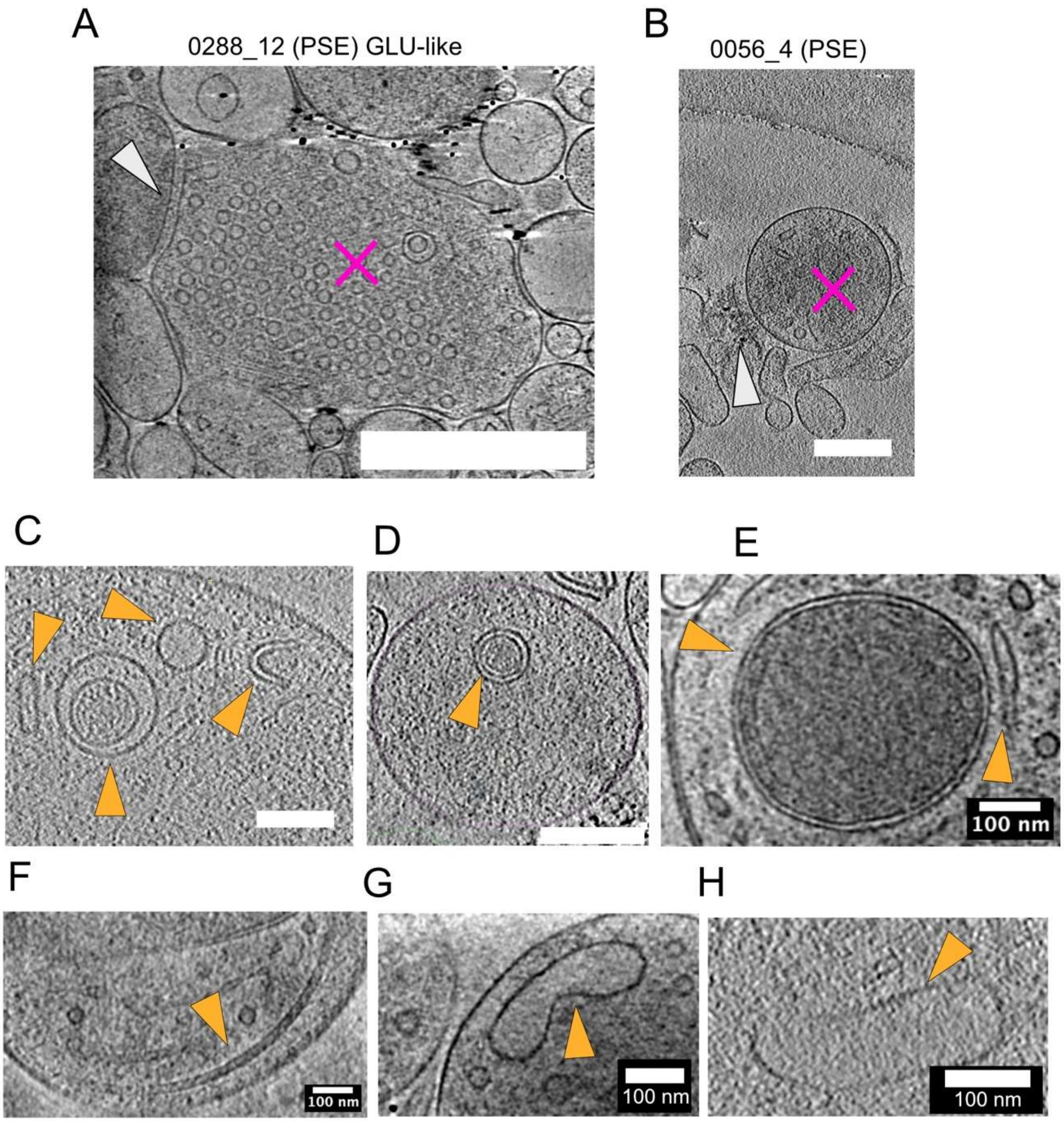

Figure S6: Images of DA with a PSD and organelles found in DA synaptosomes. A, DA synaptosome (magenta star) resembling a GLU synaptosome facing a PSE containing a PSD (grey arrow). It has 610 small synaptic vesicles, the highest observed in DA synaptosomes. Scale Bar: 500 nm. **B**, DA synaptosome (magenta star) facing an opened PSE with a PSD (grey arrow). Scale bar: 200 nm. **C**, Cytoplasmic content of a DA synaptosome containing, from left to right, a tubular vesicle, a vesicular body, a large vesicle, a C-shaped structure. Scale bar: 200 nm. **D**, A vesicular body. Scale bar: 200 nm. **E**, A mitochondrion (left) and an ER-like structure (right). Scale bar: 100 nm. **F**, A microtubule, Scale bar: 100 nm. **G,H**, Endosome-like structures. Scale bars: 100 nm.

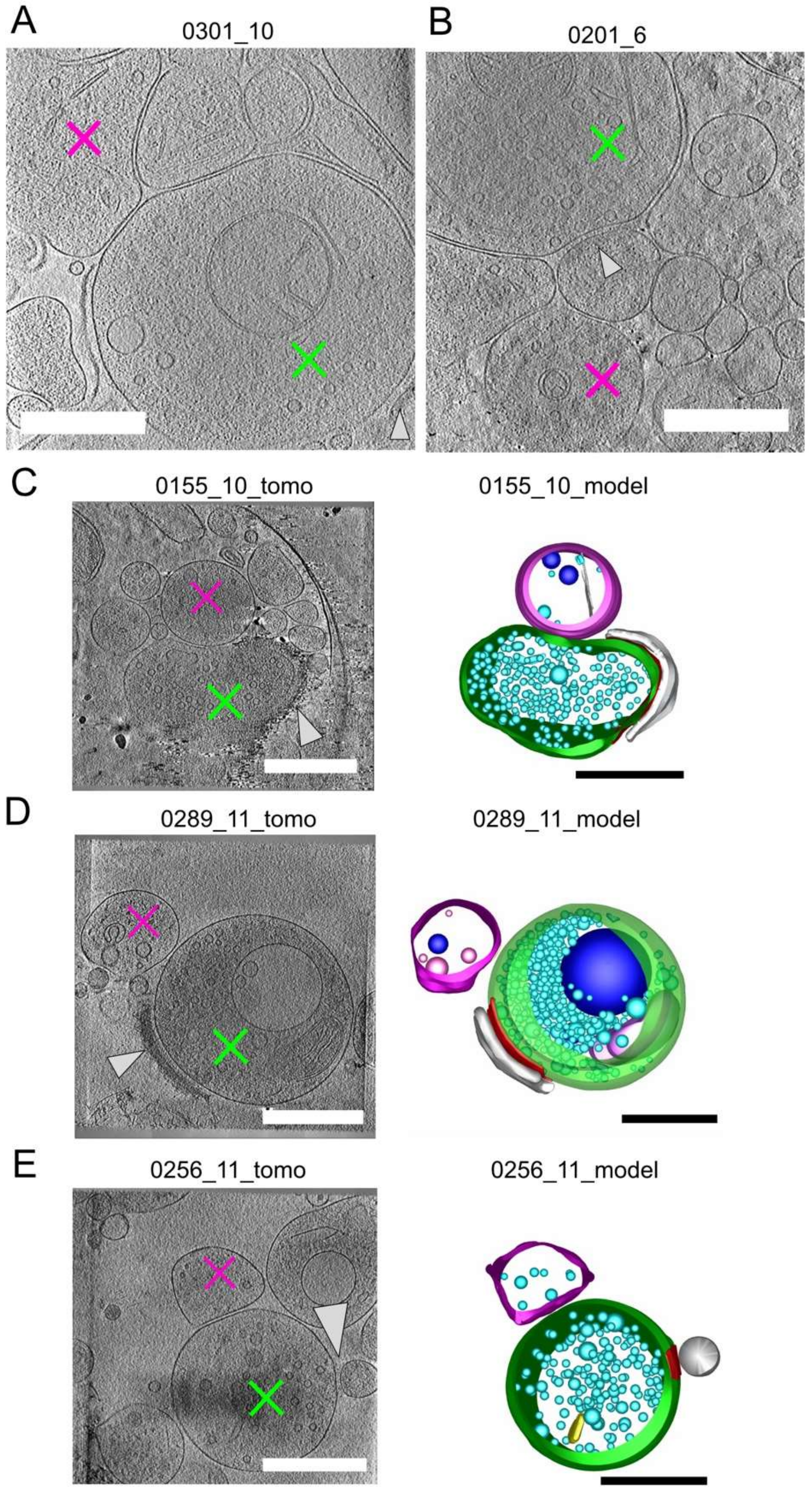

Figure S7: Gallery of reconstructed CS-DHS identified with both DA and GLU reporters. A, Single plane of the tomogram 0301_10 showing the DHS corresponding to the model in Figure 3B. DA is shown by the magenta star, GLU by the green cross and the post-synapse by the grey arrow. **B,** Single plane of the tomogram 0201_6 showing the DHS corresponding to the model in figure 3C. **C, D, E**, Examples of tomograms showing DHS (left) with their corresponding 3D model (right). Scale bars: 500 nm. Color code is similar to Figures 2 and 3.

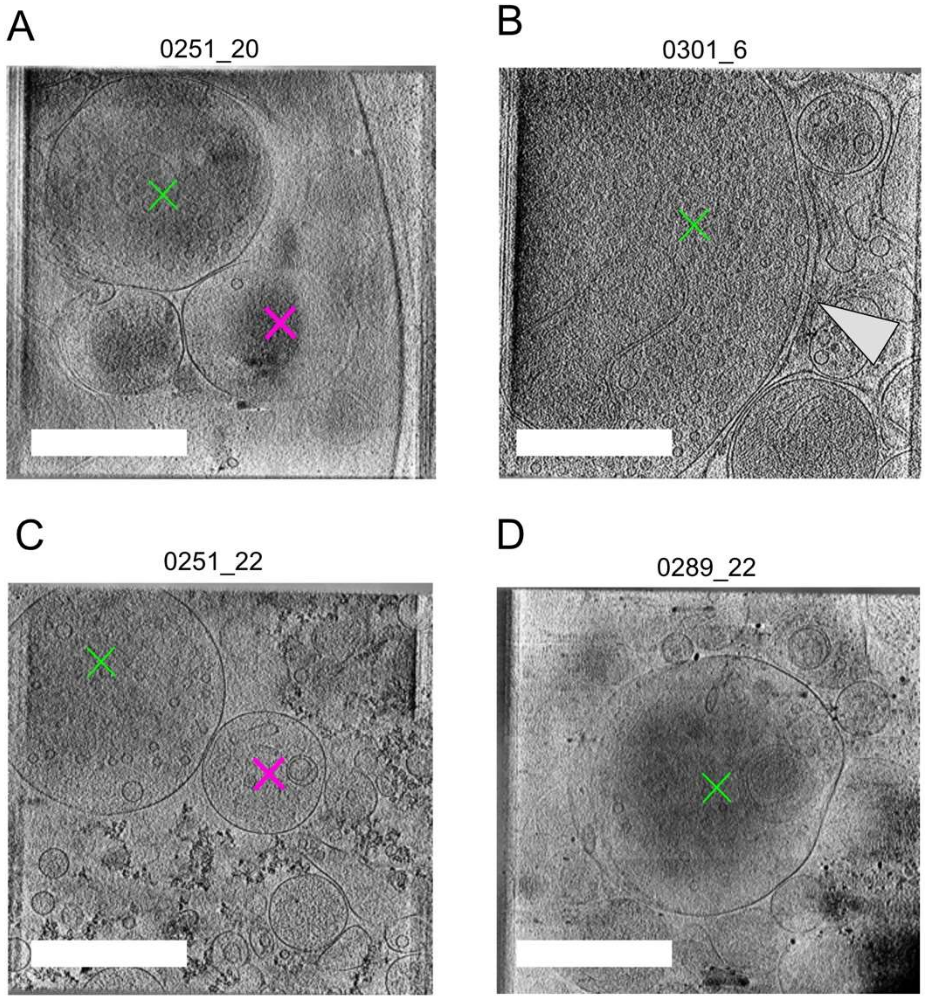

Figure S8: Examples of tomograms not retained for segmentation. A, Example of a tomogram where reconstruction resulted in a miss-alignment of the planes. **B**, Example of a tomogram plane showing a GLU synaptosome which is too big to obtain a sufficient contrast. **C**, Example of a tomogram where both DA and GLU structures are unrelated according to the correlation. **D**, Example of a tomogram for which the reconstruction failed, it results in very low information across the planes. Scale bars: 500 nm.

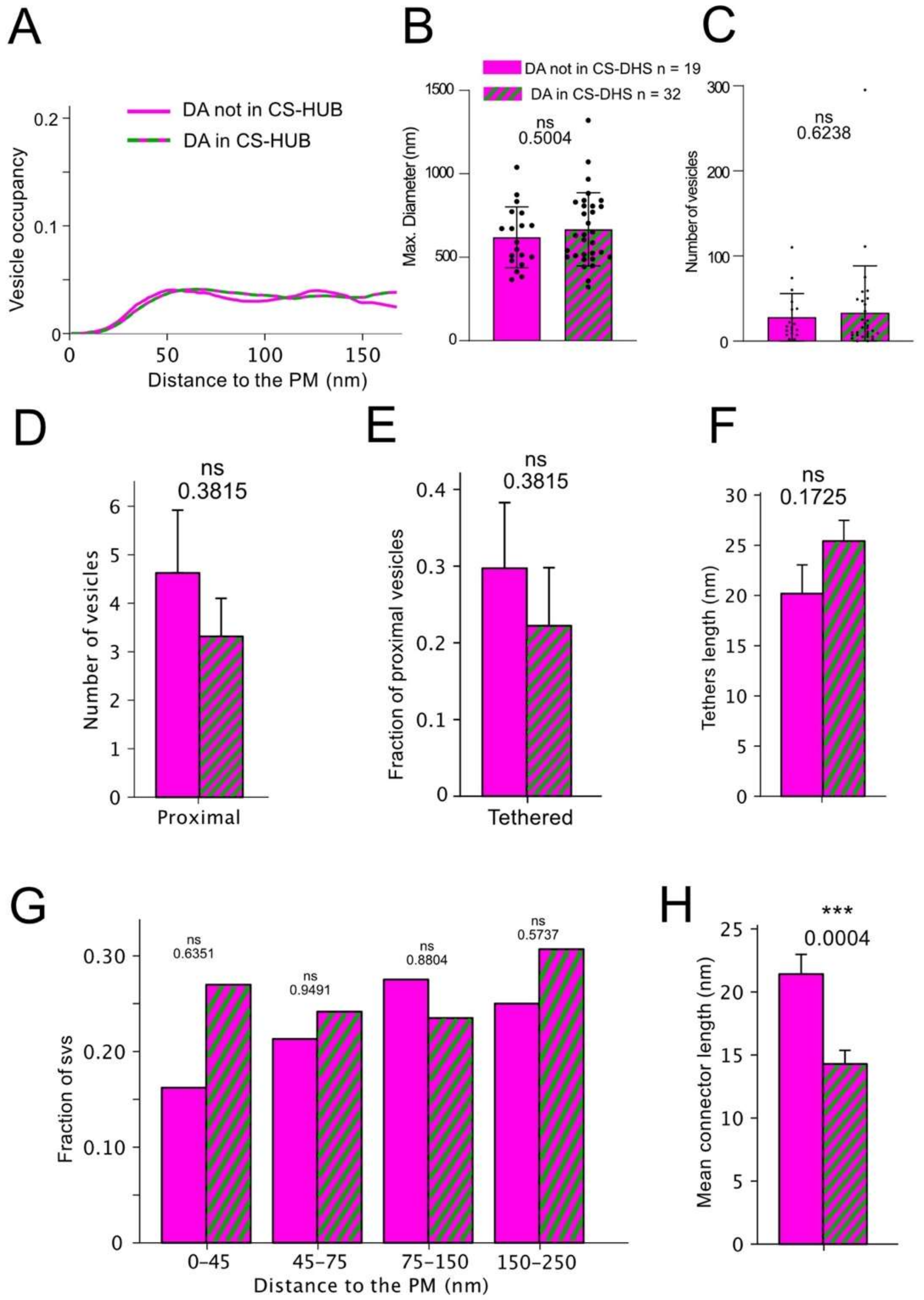

Figure S9: DA synaptosomes organization is unaffected in CS-DHS. A, Averaged fractions of the volume occupied by vesicles to the plasma membrane (PM) for DA forming CS-DHS or not. **B,** Mean maximal diameter of DA synaptosomes involved or not in CS-DHS (Mann-Whitney; p-value = 0.500). **C,** Mean number of vesicles in DA synaptosomes forming CS-DHS or not (Mann-Whitney; p-value 0.624). **D,** Averages of the number of proximal vesicles (<45 nm) from the plasma membrane (Mann-Whitney; p-value = 0.382). **E,** Averaged fractions of the volume occupied by proximal tethered vesicles (t-test; p-value 0.382). **F,** Mean tether length in DA synaptosomes involved in CS-DHS or not (t-test; p-value = 0.173). **G,** Fractions of vesicles in function of the distance to the plasma membrane (t-tests; p-values: 0 to 45 nm = 0.635; 45 to 75 nm = 0.949; 75 to 150 nm = 0.880; 150 to 250 nm = 0.574). **H,** Mean connector length of DA vesicles involved in CS-DHS or not (t-test; p-value = 0.0004).

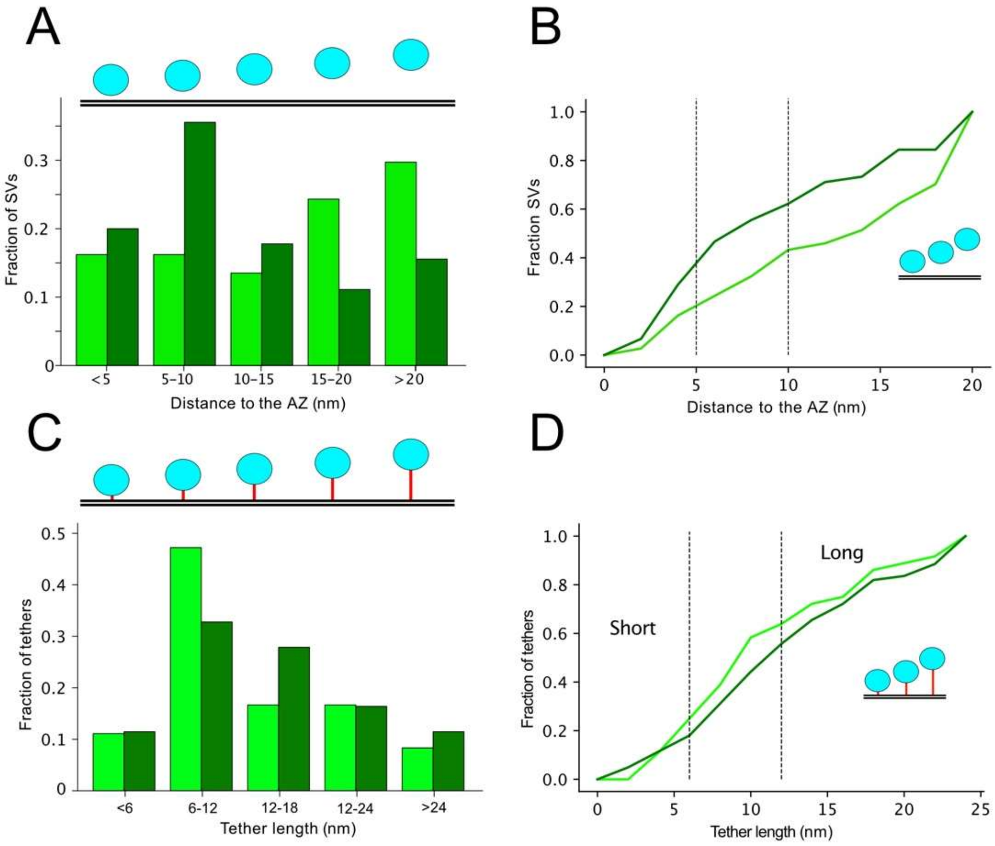

Figure S10: Distribution of proximal GLU vesicles in CS-DHS and tether length.
A, Fractions of proximal vesicles (<45 nm) localized at certain distances from the active zone shown with histograms depending on the involvement in DHS (dark green) or not (light green). **B,** Cumulative distribution of the SV distances from the active zone, data are the same as in (A). **C,** Fractions of tethers corresponding to certain lengths. Tethers from GLU involved in DHS are in dark green, the ones not involved in DHS are in light green. **D,** Cumulative distribution of the length of the tethers. Data are the same as in (C).

	TOTAL	VGLUT1-Venus	DAT-Cre +AAV	VGLUT1-Venus*DAT-Cre*Ai14 tdTomato		
**GLU**	n = 103<br>N = 14	n = 41<br>N = 4		Not in DHS<br>n = 30; N = 9	With AZ<br>n = 19; N = 8	High contrast<br>n = 16; N = 7
				In DHS<br>n = 32; N = 10	With AZ<br>n = 20; N = 10	High contrast<br>n = 10; N = 6
**DA**	n = 110<br>N = 16		n = 59*<br>N = 6*<br>*High contrast<br>n = 16<br>N = 1	Not in DHS<br>n = 30; N = 9	High contrast<br>n = 16; N = 7	> 2 SV<br>n = 8; N = 5
				In DHS<br>n = 32; N = 10	High contrast<br>n = 16; N = 7	> 2 SV<br>n = 19; N = 8

Table S1: Number of GLU and DA synaptosomes imaged across the three different models.
In total, 103 GLU synaptosomes (n) were imaged in 14 different preparations (N). Among them 62 were imaged in mice where DA synaptosomes are also identified (double-labeling: VGLUT1-venus * DAT-cre Ai 14tdTomato). Thus, we distinguished GLU synaptosomes not involved in DHS (n = 30; across 9 preparations) to GLU synaptosomes involved in DHS (n = 32; across 10 preparations). Among them, 26 synaptosomes showed sufficient information to perform Pyto analysis in both conditions. Synaptosomes where we identified the active zone (by the post-synapse) and with well contrasted membranes were retained.
In total, 110 DA synaptosomes were imaged in 16 different preparations. Among them 51 originates from the double-labeling model with 32 involved in CS-DHS and 19 not involved CS-DHS. To compare both conditions we used synaptosomes exhibiting sufficient contrast and the presence of several SV. In total, for the analysis of tethers in DA synaptosomes, we used 49 tomograms: 16 tomograms from DAT-Cre +AAV mice and 32 tomograms from VGLUT1-Venus*DAT-Cre*Ai14 tdTomato mice. After analysis, not a single synaptosome was excluded.

	With Post-synaptic element (PSE)	With mitochondrion	With multivesicular body
**GLU**	62.1 %<br>64/103	20.3 %<br>21/103	35.9 %<br>37/103
**DA**	1.8 %<br>2/110	8.2 %<br>9/110	32.7 %<br>36/110

Table S2: Proportion of GLU and DA synaptosomes with a post-synaptic element (PSE), a mitochondrion or a multivesicular body

Table S3: List of statistical tests used in this study

FIGURE	TEST	COMPARISONS	VALUES (MEAN and St. Dev.)
**Figure 2C** – *max. extent synaptosomes GLU vs. DA*	Mann-Whitney	p-value <0.0001 ****	GLU : 823.23 (STD 262.2)<br>DA : 571 (STD 182.2)
**Figure 2D** – *visible volume synaptosomes GLU vs. DA*	Mann-Whitney	p-value < 0.0001 ****	GLU : 0.0915 (STD 0.0573)<br>DA :0.0410 (STD 0.0388)
**Figure 2E** – *number of vesicles GLU vs. DA*	Mann-Whitney	p-value < 0.0001 ****	GLU: 190.8 (STD 176.4)<br>DA: 29.67 (STD 70.39)
**Figure 2F** – *Density of vesicles GLU vs. DA*	Mann-Whitney	p-value < 0.0001 ****	GLU: 1956 (STD 1166)<br>DA: 682.3 (STD 710.2)
**Figure 2H** – *CFD vesicle diameter GLU vs. DA vs. DA PSE+*	Kolmogorov-Smirnov tests	GLU vs. DA: p-value < 0.0001 ****<br><br>GLU vs. DA PSE+: p-value <0.0001****	GLU: 40.37 (STD 7.0)<br>DA: 45.10 (STD 9.7)<br>DA PSE+: 38.80 (STD 5.9)
**Figure 2I** – *CFD vesicle sphericity GLU vs. DA vs. DA PSE+*	Kolmogorov-Smirnov tests	GLU vs. DA: p-value < 0.0001 ****<br><br>GLU vs DA PSE: 0.1744 ns	GLU : 0.9871 (STD 0.02883) n = 18 897<br>DA : 0.9752 (STD 0.059) n = 2782<br>DA PSE+ : 0.9924 (STD 0.01295) n = 649
**Figure 3F** – *Mean area of AZ (GLU/PSE) or contact (DA/GLU or DA/PSE)*	Mann-Whitney	AZ vs. DA/GLU: 0.1009 ns<br><br>DA/GLU vs DA/PSE : 0.4242 ns	AZ : 0.079118 (STD 0.054978)<br>DA/GLU : 0.054103 (STD 0.03643)<br>DA/PSE : 0.062775<br>(STD 0.003880)
**Figure 3G** – *Mean cleft size of AZ (GLU/POST) or contact (DA/GLU or DA/PSE)*	Unpaired t-test<br><br><br>Mann-Whitney	AZ vs DA/GLU: <0.0001 ****<br><br>DA/GLU vs. DA/POST : 0.6630 ns	AZ : 31.82 (STD 6.237)<br>DA/GLU : 12.12 (STD 2.353)<br>DA/PSE : 13.25 (STD 3.307)
**Figure 4C** – *Percentage of proximal vesicles that are tethered GLU vs. DA*	Chi2 square	P-value <0.000.1	GLU: 0.52<br>DA: 0.25
**Figure 4D** – *Mean Tethers length GLU vs. DA*	t-test	p-value < 0.0001 ****	GLU : 13.89 (STD 7.47)<br>DA : 22.43 (STD 12.71)

**Figure 4E** – *Number of tethers in function of the distance to the AZ or PM (GLU vs. DA)*	t-tests	<5: nan<br>5-10: 0.2864 ns<br>10-20: 0.5704 ns<br>>20: 0.9191 ns	<5 GLU: 3.07 (STD 1.10)<br><5 DA: 2.00<br>5-10 GLU: 1.77 (STD 1.19)<br>5-10 DA: 1.82 (STD 1.94)<br>10-20 GLU: 0.33 (STD 0.55)<br>10-20 DA: 0.38 (STD 0.64)<br>>20 GLU: 0.11 (STD 0.32)<br>>20 DA: 0.09 (STD 0.28)
**Figure 4J** – *Percentage of all vesicles that are connected GLU vs. DA*	Chi2 test	P-value < 0.000.1	GLU: 0.52<br>DA: 0.25<br>per syn:<br>GLU: 0.53 (STD 0.33)<br>DA: 0.20 (STD 0.28)
**Figure 4K** – *Mean connector length GLU vs. DA*	t-test	p-value = <0.0001<br>****	GLU: 18.50 (STD 9.18)<br>DA: 14.91 (STD 11.06)
**Figure 4L** – *Percentage of proximal vesicles tethered or not and connected or not GLU vs. DA*			GLU T+/C+: 0.171<br>DA T+/C+: 0.064<br>GLU T+/C-: 0.366<br>DA T+/C-: 0.202<br>GLU T-/C+: 0.183<br>DA T-/C+: 0.170<br>GLU T-/C-: 0.280<br>DA T-/C-: 0.564
**Figure 5B** – *Mean number of vesicles DA T+ vs. DA T-*	Mann Whitney	P-value < 0.0001<br>****	DA T+: 47.39 (STD 34.65)<br>DA T-: 12.55 (STD 21.56)
**Figure 5C** – *Mean maximal diameter DA T+ vs. DA T-*	Mann Whitney	p-value = 0.0013<br>**	DA T+ : 731.8 (STD 206.9)<br>DA T- : 549 (STD 150.3)
**Figure 5D** – *Mean Density DA T+ vs. DA T-*	Mann Whitney	p-value = 0.0005<br>***	DA T+ : 703.9 (STD 482.3)<br>DA T- : 317.7 (STD 376.6)
**Figure 5E**– *Number of proximal vesicle per μm^2 in function of the distance to the plasma membrane*	t-tests	0-5: > 0.99<br>5-10 = 0.0038 **<br>10-15 = 0.017 *<br>15-20 = 0.0056 **<br>20-45 = 0.0914 ns	0-5 T+: 0.17<br>0-5 T-: nan<br>5-10 T+: 3.05 (STD 5.62)<br>5-10 T-: 0.14 (STD 0.61)<br>10-15 T+: 3.95 (STD 5.69)<br>10-15 T-: 0.79 (STD 1.95)<br>15-20 T+: 5.16 (STD 8.29)<br>15-20 T-: 1.11 (STD 2.56)<br>20-45 T+: 3.64 (STD 3.68)<br>20-45 T-: 2.77 (STD 6.60)
**Figure 5F**– *CFD vesicle diameter T+ vs. all*	Kolmogorov-Smirnov	p-value = 0.0016<br>**	
**Figure 5G**– *CFD sphericity vesicle T+ vs. all*	Kolmogorov-Smirnov	p-value < 0.0001<br>****	

**Figure 5K**– *Nearest neighbor distance T+ DA vs. GLU*	Mann-Whitney	p-value < 0.0001<br>****	DA : 216.1 (STD 193.6)<br>GLU : 100.4 (STD 72 .8)
**Figure 6C** – *Mean number of vesicles GLU DA – vs. GLU DA+*	Mann Whitney	p-value = 0.8640<br>ns	GLU DA-: 205.7 (STD 195.3)<br>GLU DA+: 222.9 (STD 214.5)
**Figure 6D** – *Mean density of vesicle in GLU DA+ vs. GLU DA-*	Mann Whitney	p-value = 0.5768<br>ns	GLU DA+: 1943 (STD 1314)<br>GLU DA-: 1893<br>(STD 772.9)
**Figure 6E** – *Mean active zone area GLU DA+ vs. GLU DA-*	t-test	p-value = 0.5512<br>ns	GLU DA-: 0.06 (STD 0.03)<br>GLU DA+: 0.07 (STD 0.04)
**Figure 6F** – *Vesicle occupancy from the AZ between GLU DA+ and GLU DA-*<br><br>*Histogram of binned values not shown*	t-test, binned values	0-45 = 0.0215 *<br>75-150 = 0.0195 *<br>150-250 = 0.0016 **	GLU DA-: 0.03 (STD 0.02)<br>GLU DA+: 0.05 (STD 0.03)<br>GLU DA-: 0.10 (STD 0.02)<br>GLU DA+: 0.07 (STD 0.02)<br>GLU DA-: 0.11 (STD 0.02)<br>GLU DA+: 0.08 (STD 0.02)
**Figure 6H** - *Percentage of proximal vesicles that are tethered GLU DA- vs. GLU DA+*	Chi2 test	p-value = 0.2041<br>ns	DA- GLU : 0.47<br>DA+ GLU : 0.61<br><br>per syn :<br>GLU DA-: 0.31 (STD 0.38)<br>GLU DA+: 0.52 (STD 0.33)
**Figure 6I** – *Number of tethers in function of the distance to the AZ on GLU DA- vs. GLU DA+*	t-tests	<5: 0.4638 ns<br>5-10: 0.5421 ns<br>>10: 0.4816 ns	<5 GLU DA-: 3.33 (STD 1.63)<br><5 GLU DA+: 2.89 (STD 0.60)<br>5-10 GLU DA-: 1.83 (STD 0.75)<br>5-10 GLU DA+: 2.15 (STD 1.14)<br>>10 GLU DA-: 1.00 (STD 0.0)<br>>10 GLU DA+: 1.20 (STD 0.45)
**Figure 6J** – *Percentage of proximal vesicles that are connected*	Chi2 test	p-value = 0.0183<br>*	DA- GLU :0.27<br>DA+ GLU :0.47<br><br>GLU DA-: 0.216 (STD 0.38 ; SEM 0.10)<br>GLU DA+: 0.467 (STD 0.30 ; SEM 0.09)
**Figure 6K** – *Percentage of proximal vesicles tethered or not and connected or not in GLU DA- vs. GLU DA+*	Chi-squared test	p-value = 0.0435<br>*	T+/C+ GLU DA-: 0.0054<br>T+/C+ GLU DA+: 0.0267<br>T+/C- GLU DA-: 0.405<br>T+/C- GLU DA+: 0.333<br>T-/C+ GLU DA-: 0.162<br>T-/C+ GLU DA+: 0.200<br>T-/C- GLU DA-: 0.378<br>T-/C- GLU DA+: 0.200

**Figure S1B** – *Loading capacity of FM4-64*	Mann Whitney	p-value < 0.0001 ****	KCl: 2.136 (STD 3.960)<br>Ctl: 1.073 (STD 3.513)
**Figure S1C** – Release capacity of FM4-64	Mann Whitney	p-value < 0.0001 ****	KCl: 2.102 (STD 1.865)<br>Ctl: 0.9745 (STD 1.497)
**Figure S9B** – *Mean maximal diameter DA in CS-DHS vs. DA not in CS-DHS*	Mann-Whitney	p-value = 0.5004 ns	DA not in CS-DHS: 619.6 (STD 182.9)<br>DA in CS-DHS: 667.5 (STD 219.2)
**Figure S9C** – *Mean number of vesicles DA in CS-DHS vs. DA not in CS-DHS*	Mann-Whitney	p-value = 0.6238 ns	DA not in CS-DHS: 28.00 (STD 27.92)<br>DA in CS-DHS: 33.39 (STD 54.98)
**Figure S9D** – *Mean number of proximal vesicles*	t-test	p-value = 0.3815 ns	DA not in CS-DHS: 4.62 (STD 3.66)<br>DA in CS-DHS: 3.32 (STD 3.42)
**Figure S9E** – *Fraction of proximal vesicles tethered*	t-test	p-value = 0.3815 ns	DA not in CS-DHS: 0.297 (STD 14.03)<br>DA in CS-DHS: 0.222 (STD 0.29)
**Figure S9F** – *mean tether length*	t-test	p-value = 0.1725 ns	DA not in CS-DHS: 20.19 (STD 0.23)<br>DA in CS-DHS: 25.42 (STD 8.76)
**Figure S9G** – *Fractions of vesicles in function of the distance to the PM*	t-tests	0-45: p-value = 0.6351 ns<br>45-75: p-value 0.9491 ns<br>75-150: p-value = 0.8804 ns<br>150-250: p-value = 0.5737 ns	0-45 DA not in CS-DHS: 0.01 (STD 0.01)<br>0-45 DA in CS-DHS: 0.01 (STD 0.01)<br>45-75 DA not in CS-DHS: 0.04 (STD 0.03)<br>45-75 DA in CS-DHS: 0.04 (STD 0.03)<br>75-150 DA not in CS-DHS: 0.03 (STD 0.02)<br>75-150 DA in CS-DHS: 0.04 (STD 0.02)<br>150-250 DA not in CS-DHS: 0.03 (STD 0.03)<br>150-250 DA in CS-DHS: 0.04 (STD 0.02)
**Figure S9H** – *Mean connector length*	t-test	p-value 0.0004 ***	DA not in CS-DHS: 21.42 (STD 11.68)<br>DA in CS-DHS: 14.29 (STD 13.01)
